# MOMENTARY INTERACTIONS WITH FRIENDS AND CARE RECIPIENTS AND CARDIOVASCULAR REACTIVITY IN DEMENTIA CAREGIVERS

**DOI:** 10.1093/geroni/igae098.1886

**Published:** 2024-12-31

**Authors:** Yee To Ng

**Affiliations:** University of Michigan, Ann Arbor, Michigan, United States

## Abstract

Friends may serve as an important resource for dementia caregivers who often experience stress and heightened cardiovascular risk. This study examines momentary associations between friend interactions and cardiovascular reactivity, and tests whether friend interactions moderate the association between interaction with the care recipients (CRs; both positive and negative) and cardiovascular reactivity among Black and White dementia caregivers. Using Ecological Momentary Assessments (EMA), 159 dementia caregivers (Mean age: 62.24, 60% White) reported interactions with CRs (including time spent and levels of positivity and negativity) and indicated any friend interactions every 3 hours over five days. ECG monitors collected real-time physiological data (including heart rate [HR] and heart rate variability [HRV-RMSSD]). Findings revealed no racial differences in contact frequency with friends. Although Black caregivers spent less time with CRs, they reported similar levels of positive and negative interactions with CRs than White caregivers. While positive interactions with CRs were not associated with HR or HRV among caregivers, negative interactions with CRs were linked to momentary increases in HR, observed only among Black caregivers. Moreover, friend interactions were linked to momentary elevation in HR and reduction in HRV for both Black and White caregivers. However, friend interactions did not moderate the effect of negative interactions with CRs on cardiovascular reactivity. This study highlights racial disparities in the amount of time spent with the care recipients and its momentary effects on cardiovascular health. Friend interactions may not alleviate the impact of caregiving on cardiovascular burden but remain linked to momentary cardiovascular health.

